# Edaphic Niche Differentiation Exceeds Field Ecophysiological Divergence Among Wild *Rosa* Species Across the Mountain Systems of Eastern Kazakhstan

**DOI:** 10.3390/biology15151247

**Published:** 2026-07-28

**Authors:** Anar Myrzagaliyeva, Sultan Kauanov, Shynar Tustubayeva, Serik Irsaliyev, Moldir Sharipova, Aidyn Orazov

**Affiliations:** Laboratory “NatureLab”, Astana International University, 8 Kabanbay Batyr Avenue, Astana 010000, Kazakhstan; an.myrzagaliyeva@gmail.com (A.M.); sultan.kauanov96@gmail.com (S.K.); sirsaliyev@gmail.com (S.I.); moldirzhumagul@gmail.com (M.S.)

**Keywords:** edaphic niche differentiation, environmental filtering, *Rosa acicularis*, *Rosa alberti*, *Rosa spinosissima*, chlorophyll fluorescence, functional trait convergence, mountain ecology, plant–soil relationships, Kazakhstan

## Abstract

Environmental heterogeneity can promote niche differentiation among closely related species, yet ecological segregation may not be mirrored by traits measured during a single field campaign. We tested whether edaphic differentiation exceeded morphological and ecophysiological divergence in *Rosa acicularis*, *R. alberti*, and *R. spinosissima* across the Altai and Tarbagatai Mountains of eastern Kazakhstan. The dataset comprised 40 field records representing 25 geographic coordinates and 30 species–site combinations. Plant height, crown width, chlorophyll fluorescence, chlorophyll-related outputs, and soil pH, electrical conductivity, total nitrogen, and total carbon were analysed using FDR-controlled univariate tests with Dunn post hoc comparisons, PERMANOVA, reduced-variable PCA, partial redundancy analysis, correlation analysis, and small-sample explanatory models with leave-one-out validation. Within Tarbagatai, species identity explained 48.6% of multivariate soil variation but only 9.2% of plant-trait variation. Omnibus differences in pH, nitrogen, and carbon remained significant after correction, and the species–site analysis also supported EC differentiation. The soil block explained no significant independent component of plant-trait variation after species and mountain system were taken into account. *Rosa spinosissima* was taller in the Altai at the observation level, and a species-plus-region model showed moderate cross-validated performance for height; models for other traits had little predictive value. These results support edaphic niche differentiation accompanied by limited divergence in field-measured traits. Because the study was observational and temporally restricted, the findings demonstrate environmental association rather than adaptation. Still, they identify testable hypotheses and support environmentally stratified conservation of wild *Rosa* genetic resources.

## Simple Summary

Closely related plant species can occupy contrasting soil environments without displaying equally pronounced differences in traits measured during a single field survey. We compared three wild *Rosa* species from the Altai and Tarbagatai Mountains of eastern Kazakhstan using plant size, chlorophyll fluorescence, chlorophyll-related indices, and soil chemistry. The species differed strongly in soil pH, electrical conductivity, total nitrogen, and total carbon, whereas most morphological and physiological traits overlapped. *Rosa spinosissima* also showed a marked regional difference in plant height. These findings show that edaphic niche differentiation may be more evident than short-term physiological divergence in natural populations and support environmentally stratified conservation sampling and controlled follow-up experiments.

## 1. Introduction

Mountain ecosystems exhibit pronounced environmental heterogeneity over short spatial distances. Variation in elevation, temperature, moisture availability, substrate properties, solar exposure, and disturbance creates complex habitat mosaics that can promote ecological differentiation among closely related plant species [1,2,3]. These systems provide natural settings for examining how species partition environmental space and persist under contrasting ecological conditions, an issue of growing importance because mountain vegetation is sensitive to climate change, land-use pressure, erosion, and altered soil nutrient dynamics [4,5,6].

Ecological differentiation among related taxa may arise through genetically based adaptation, phenotypic plasticity, dispersal limitation, biotic interactions, or historical constraints [7,8,9]. A central question in functional ecology is whether environmental segregation is accompanied by divergence in measurable traits. Plant height and crown width integrate cumulative growth and competitive conditions, whereas chlorophyll fluorescence and pigment-related indices provide complementary snapshots of photosynthetic state [10,11,12,13,14,15,16,17,18]. Strong environmental differentiation may nevertheless coexist with overlapping field traits when species maintain similar realised functional states through acclimation, compensatory trait combinations, or microsite selection.

The genus *Rosa* L. is well suited to testing this relationship. Wild roses are ecologically widespread and taxonomically complex shrubs of forest margins, river valleys, shrublands, mountain meadows, rocky slopes, and steppes across temperate Eurasia [19,20,21]. Hybridisation, polyploidy, apomixis, introgression, and morphological plasticity can increase within-species variability and obscure ecological differences [22,23,24]. *Rosa acicularis* is commonly associated with relatively moist forest-edge and valley habitats, *R. alberti* with mountain and foothill environments of Central Asia, and *R. spinosissima* with a broader range of open, rocky, shrub, and steppe habitats. Wild *Rosa* populations also contribute to slope stabilisation and habitat structure and represent genetic resources for conservation, breeding, horticulture, and restoration [25,26,27].

Eastern Kazakhstan contains the Altai and Tarbagatai mountain systems, which differ in climate, vegetation structure, soil development, and biogeographic history. Both regions support natural populations of *R. acicularis*, *R. alberti*, and *R. spinosissima*, but the extent of their ecological differentiation remains poorly quantified. It is unclear whether the species are separated primarily by the edaphic conditions they occupy or whether consistent morphological and photosynthetic differences accompany that segregation.

Soil pH, electrical conductivity (EC), total nitrogen (TN), and total carbon (TC) describe complementary dimensions of the edaphic environment. Soil pH affects nutrient solubility, microbial activity, and root acquisition; EC integrates soluble-ion concentration; and TN and TC broadly reflect nutrient and organic-matter status [28,29,30,31,32]. These variables often covary, particularly TN and TC, so multivariate and correlation-based analyses are needed to distinguish integrated environmental gradients from apparently independent effects.

Previous research on wild roses has focused mainly on taxonomy, morphology, genetic diversity, fruit chemistry, medicinal value, and potential distribution [33]. Simultaneous field assessment of plant traits and local soil conditions remains scarce in the Central Asian mountains. Such evidence has practical value: conservation collections based solely on geographic distance may overlook populations inhabiting contrasting soil environments, thereby underrepresenting ecological and genetic variation.

This study evaluated the relative contributions of edaphic, geographic, and species-level factors to morphological and ecophysiological variation in *R. acicularis*, *R. alberti*, and *R. spinosissima* from the Altai and Tarbagatai Mountains. Four a priori predictions were tested: (1) species differentiation would be stronger in the multivariate soil environment than in the plant-trait matrix; (2) Altai–Tarbagatai contrasts would be species specific; (3) apparent plant–soil relationships would weaken after spatial aggregation and correction for multiple testing; and (4) parsimonious species-and-region models would outperform models augmented with soil and coordinate predictors.

## 2. Materials and Methods

### 2.1. Study Area, Target Species, and Sampling Design

**Figure 1:**
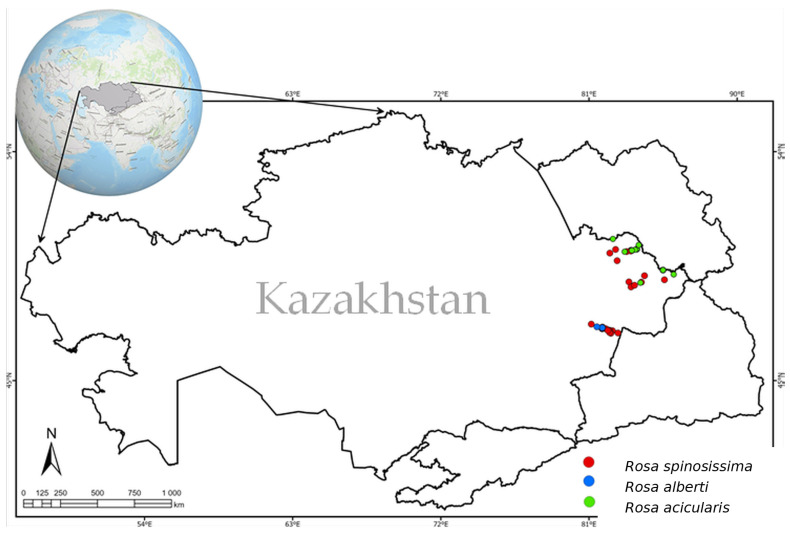
Geographic distribution of field records for *Rosa acicularis*, *R. alberti*, and *R. spinosissima* in Kazakhstan. Sampling was concentrated in the Altai and Tarbagatai mountain systems of eastern Kazakhstan.

Fieldwork was conducted from June to July 2024 in the Altai and Tarbagatai Mountains of eastern Kazakhstan (Figure 1). Altai localities were situated at 50.32–50.53° N and 82.62–84.02° E, whereas Tarbagatai localities were located at 47.17–47.26° N and 81.48–81.81° E. The study targeted naturally occurring *R. acicularis* Lindl., *R. alberti* Regel, and *R. spinosissima* L., identified in the field based on diagnostic vegetative and reproductive characters, using regional floristic treatments. Coordinates were recorded in WGS 84.

The survey produced archived field records linking species identity, locality, collection date, morphology, fluorescence, chlorophyll status, and soil chemistry. Some records shared coordinates and could not be treated as fully independent spatial replicates. Therefore, observation records were retained for descriptive and primary univariate analyses. In contrast, records sharing the same species and coordinate were averaged to form species–site units for spatially conservative sensitivity, multivariate correlation, and modelling analyses. The unequal representation of species and mountain systems reflected natural occurrence and field accessibility rather than a balanced experimental design.

### 2.2. Morphological Measurements

Plant height and crown width were recorded in centimetres. Height was measured from the soil surface to the uppermost part of the shrub, and crown width represented the horizontal extent of the above-ground crown. These traits were selected as integrative indicators of plant stature, cumulative growth, resource availability, and competitive environment [34,35]. Each archived value was treated as a single field observation; no unverified biological or technical replicates were reconstructed.

### 2.3. Chlorophyll Fluorescence and Chlorophyll Status

Chlorophyll fluorescence was measured in situ using an OS-30p+ portable pulse-modulated fluorometer (Opti-Sciences, Hudson, NH, USA). Minimum fluorescence (Fo) and maximum fluorescence (Fm) were recorded, variable fluorescence was calculated as Fv = Fm − Fo, and Fv/Fm and Fv/Fo were calculated as (Fm − Fo)/Fm and (Fm − Fo)/Fo, respectively [14,15,16,36].

Leaf chlorophyll status was assessed with a CCM-300 Chlorophyll Content Meter (Opti-Sciences, Hudson, NH, USA). The instrument reports the chlorophyll fluorescence ratio CFR = F735/F700 and an internally calibrated estimate of chlorophyll content (mg m^−2^) [37]. CFR and estimated chlorophyll were treated as paired instrument outputs rather than independent physiological measurements.

### 2.4. Soil Sampling and Laboratory Analysis

Soil associated with the upper root zone near the examined shrubs was air-dried, cleared of coarse material, gently disaggregated, passed through a 2 mm sieve, and homogenised [38,39]. Soil pH was measured potentiometrically in an aqueous suspension using an AquaSearcher AB23PH-*F* bench meter with an ST320 electrode (OHAUS Corporation, Parsippany, NJ, USA). EC was measured conductometrically in an aqueous extract and expressed in µS cm^−1^ [30,40]. TN and TC were determined by high-temperature dry combustion with a CN/CHNS elemental analyser following the general principles of ISO 13878 and ISO 10694 [41,42]. All samples were processed using a common laboratory workflow. The exact model of the CN/CHNS elemental analyser was not preserved in the archived laboratory record.

### 2.5. Statistical Analysis

#### 2.5.1. Data Screening and Analytical Units

Before analysis, variables were checked for missing values, inconsistent units, implausible observations, and duplicated species–coordinate combinations. No values were imputed. The two analytical resolutions served different purposes: the 40 observation records retained the information obtained during fieldwork, while the 30 species–site means reduced the influence of repeated coordinates and potential pseudoreplication [43,44]. Agreement in the direction across resolutions was used as a robustness criterion.

#### 2.5.2. Record-Level Univariate Analyses

Species and mountain system summarised final sample sizes and descriptive statistics. Because *R. alberti* occurred only in Tarbagatai, interspecific comparisons were restricted to Tarbagatai, where all three species were represented. Each response was first tested with a Kruskal–Wallis rank-sum test [45]. Significant omnibus tests were followed by Dunn’s post hoc comparisons with Benjamini–Hochberg adjustment [46,47]. Regional contrasts were evaluated separately for *R. acicularis* and *R. spinosissima* with two-sided Mann–Whitney tests [48]. Kruskal–Wallis effects were summarised using epsilon-squared, and two-group contrasts were summarised using rank-biserial correlations. False-discovery-rate control was applied within prespecified families of omnibus, regional, and correlation tests; *q* < 0.05 was considered statistically supported.

#### 2.5.3. Species–Site Analyses

The major univariate comparisons were repeated using species–site means. Standardised plant traits (height, crown width, Fv/Fm, CFR, and estimated chlorophyll) and standardised soil variables (pH, EC, TN, and TC) were analysed separately by Euclidean-distance PERMANOVA with 9999 permutations [49]. PERMDISP was used to distinguish centroid separation from differences in within-group dispersion [50]. Species were compared within Tarbagatai, and mountain systems were compared separately within *R. acicularis* and *R. spinosissima*.

Spearman correlations among the ten recorded variables were used for two explicit purposes: to diagnose covariance and measurement redundancy and to determine whether simple plant–soil associations remained after spatial aggregation and false-discovery-rate correction. All 45 unique pairwise tests were adjusted simultaneously, and the complete structure was displayed as a lower-triangular heat map.

PCA was used as an exploratory tool for summarising joint variation [51]. To prevent highly redundant variables from dominating the ordination, Fv/Fo was excluded because it is algebraically linked to Fv/Fm, estimated chlorophyll was excluded because it is derived from CFR, and TC was excluded because it was almost perfectly correlated with TN. The revised PCA, therefore, comprised height, crown width, Fv/Fm, CFR, pH, EC, and TN. Partial redundancy analysis tested the conditional association between the plant-trait matrix and the four-variable soil block after species identity and mountain system were included as conditioning variables [52,53].

### 2.6. Geographic and Edaphic Explanatory Models

Explanatory models were fitted to the 30 species–site units. Response variables were standardised. Latitude and longitude were centred within each mountain system and converted to local northing and easting offsets; these terms were interpreted only as proxies for residual within-region spatial structure, not as direct environmental drivers [54,55].

A composite edaphic gradient (soil PC1) was derived from standardised pH, EC, TN, and TC. It explained 58.9% of soil variation and increased with EC, TN, and TC while decreasing with pH. Four candidate linear models were specified a priori for each plant response: Base (species + mountain system), Soil (Base + soil PC1), Spatial (Base + local northing + local easting), and Full (Base + soil PC1 + local coordinates). Models were ranked using AICc and Akaike weights [56,57]; coefficient uncertainty was estimated with HC3 robust standard errors [58], and predictive performance was assessed by leave-one-out cross-validated R^2^ [59]. Interactions and higher-order terms were excluded to limit overfitting. All statistical analyses and figures were produced in Python 3 (Python Software Foundation, Wilmington, DE, USA).

## 3. Results

### 3.1. Sampling Coverage and Descriptive Variation

The final dataset contained 40 observation records from 25 unique coordinates and 30 species–site combinations (Table 1). *R. spinosissima* was most frequently represented (*n* = 21), followed by *R. acicularis* (*n* = 15) and *R. alberti* (*n* = 4). All three species occurred in Tarbagatai, whereas only *R. acicularis* and *R. spinosissima* were sampled in the Altai. Mean Fv/Fm values were similar among species, while *R. alberti* showed the lowest descriptive EC, TN, and TC values (Table 2).

### 3.2. Record-Level Univariate Comparisons

Within Tarbagatai, observation-level Kruskal–Wallis tests identified FDR-supported interspecific differences in soil pH (*H* = 9.99, *q* = 0.023), TN (*H* = 12.11, *q* = 0.012), and TC (*H* = 12.09, *q* = 0.012); EC was nominally significant but did not pass the across-variable FDR threshold (*H* = 7.62, *q* = 0.055). No morphological, fluorescence, or pigment variable differed significantly among species (Table 3). Dunn’s post hoc tests confirmed lower pH for *R. acicularis* than for the other two species, lower EC for *R. alberti* than for *R. acicularis* and *R. spinosissima*, and higher TN and TC for *R. acicularis* than for *R. alberti*. Observation-level TN and TC contrasts between *R. acicularis* and *R. spinosissima* were also supported, whereas the *R. alberti*–*R. spinosissima* nutrient contrasts were weaker.

Regional contrasts were species-specific. *R. spinosissima* was taller in the Altai than in Tarbagatai at the observation level (median 126.3 vs. 68.1 cm; *U* = 90.0, *q* = 0.025; rank-biserial *r* = 0.837). The same direction was retained after aggregation (126.3 vs. 71.7 cm), but the contrast was no longer significant after correction (*q* = 0.364). No regional univariate contrast was significant for *R. acicularis*.

### 3.3. Species–Site Post Hoc and Multivariate Patterns

**Figure 2:**
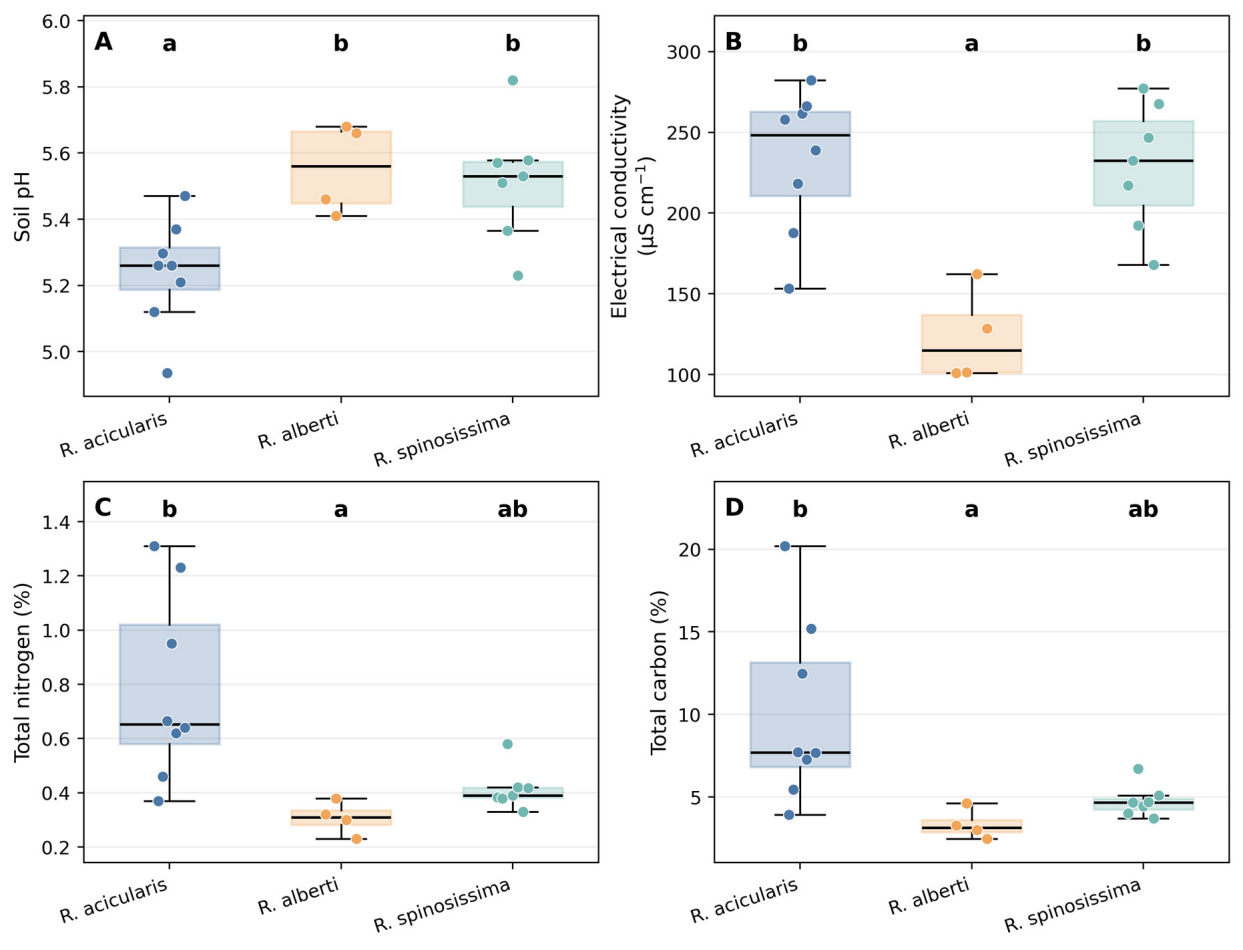
Soil differentiation among *Rosa* species in Tarbagatai based on species–site means: (**A**) soil pH; (**B**) electrical conductivity; (**C**) total nitrogen; and (**D**) total carbon. Boxes show medians and interquartile ranges, and points represent species–site combinations. Blue, orange, and green denote *R. acicularis*, *R. alberti*, and *R. spinosissima*, respectively. Within each panel, groups sharing a letter did not differ in Dunn post hoc comparisons after Benjamini–Hochberg adjustment.

The species–site sensitivity analysis retained significant omnibus differences in pH, EC, TN, and TC (all *q* ≤ 0.037; Table 3). Dedicated Dunn comparisons refined the pairwise interpretation: *R. acicularis* differed from both *R. alberti* and *R. spinosissima* in pH; *R. alberti* differed from both species in EC; and the strongest TN and TC contrast was between *R. acicularis* and *R. alberti* (Table 4). The *R. acicularis*–*R. spinosissima* TN and TC comparisons remained directional but did not pass the within-variable Dunn adjustment. Significance letters in Figure 2 summarise these site-level post hoc results.

At the species–site level, the five-variable plant-trait matrix did not differ among species within Tarbagatai (PERMANOVA *F* = 0.81, *p* = 0.5761, R^2^ = 0.092), whereas the four-variable soil matrix showed strong differentiation (*F* = 7.57, *p* = 0.0005, R^2^ = 0.486; Table 5). Soil dispersion also differed (PERMDISP *p* = 0.0443), indicating that the result reflected both centroid separation and unequal within-species environmental variability. The combined matrix was significant (*F* = 2.91, *p* = 0.0015, R^2^ = 0.267), with no evidence of unequal dispersion.

Regional multivariate patterns also differed by species (Table 5). In *R. acicularis*, the soil matrix differed between the Altai and Tarbagatai (R^2^ = 0.263, *p* = 0.0330), whereas the plant-trait matrix did not. In *R. spinosissima*, the plant-trait matrix differed between regions (R^2^ = 0.203, *p* = 0.0272), but the soil-centroid test was not significant. Soil dispersion in *R. spinosissima* differed strongly between regions (*p* = 0.0034), so its regional soil contrast should not be interpreted as a uniform shift.

### 3.4. Reduced-Variable PCA and Conditional Plant–Soil Association

**Figure 3:**
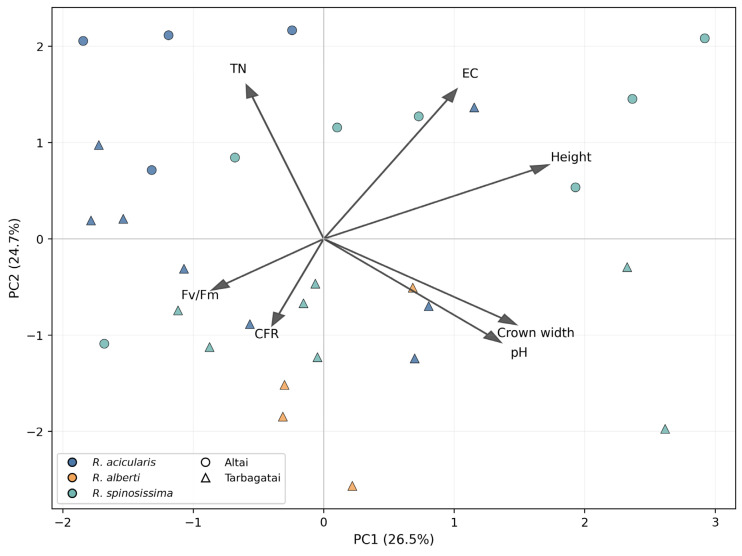
PCA biplot based on 30 species–site means and seven standardised, non-redundant variables. Estimated chlorophyll, Fv/Fo, and TC were excluded because of their dependence on CFR, Fv/Fm, and TN, respectively. Point colour identifies species, and point shape identifies the mountain system.

After removal of the three redundant variables, the first three PCA axes explained 68.5% of standardised variation (PC1 = 26.5%, PC2 = 24.7%, and PC3 = 17.3%; Figure 3). PC1 was associated mainly with plant height, crown width, and pH; PC2 contrasted higher EC and TN with lower pH and CFR; and CFR dominated PC3. The revised ordination therefore separated morphological, edaphic, and pigment-related structure without allowing the near-duplicate TN–TC and CFR–chlorophyll vectors to dominate.

In partial redundancy analysis, the four-variable soil block did not explain a significant independent component of the multivariate plant-trait matrix after species and mountain system were conditioned out (partial R^2^ = 0.127, *F* = 0.80, *p* = 0.649). Thus, strong species-level soil separation was not accompanied by a simple conditional linear response in the measured plant traits.

### 3.5. Correlation Structure

**Figure 4:**
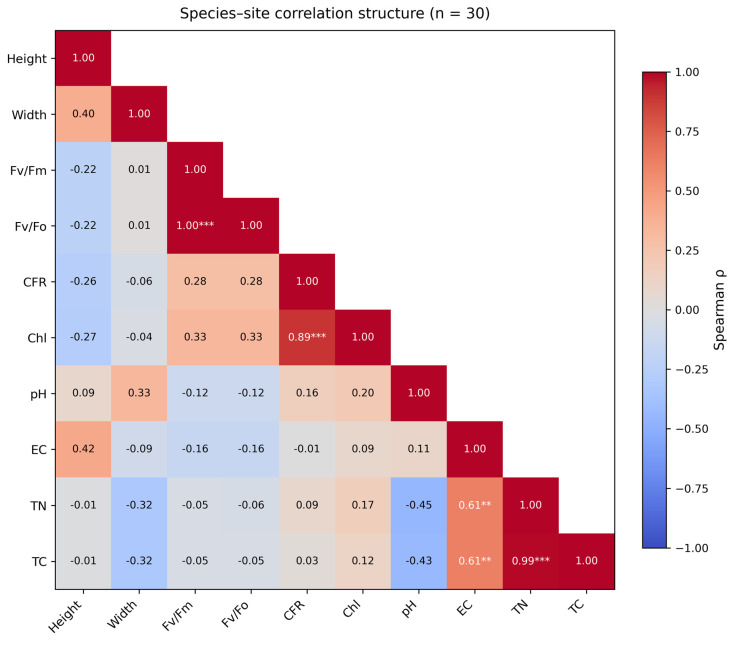
Spearman correlation heat map for ten morphological, physiological, and edaphic variables based on 30 species–site means. Cell values are Spearman coefficients; asterisks indicate Benjamini–Hochberg FDR-adjusted significance (** *q* < 0.01, *** *q* < 0.001). Chl denotes estimated chlorophyll; EC, electrical conductivity; TN, total nitrogen; and TC, total carbon.

The correlation analysis was retained as a diagnostic of covariance and potential one-to-one plant–soil coupling. At the species–site level, it identified two clear dependency modules (Figure 4; Table 6): TN and TC were almost perfectly correlated (ρ = 0.989, *q* < 0.001), and both covaried positively with EC (ρ = 0.611–0.613, *q* = 0.003); CFR was strongly related to the instrument-derived chlorophyll estimate (ρ = 0.892, *q* < 0.001). In contrast, correlations between plant size or Fv/Fm and individual soil variables did not remain significant after FDR correction.

### 3.6. Geographic and Edaphic Models of Plant Traits

The complete candidate-model comparison is presented in Table 7. For plant height, the Base model containing species and mountain system received the strongest support (Akaike weight = 0.748; adjusted R^2^ = 0.376; LOO R^2^ = 0.294). Adding soil PC1 or local coordinate offsets increased AICc and did not improve cross-validated prediction.

The remaining responses showed weak predictive structure. The Base model ranked first for crown width, CFR, and chlorophyll, but their LOO R^2^ values were −0.090, −0.039, and 0.023, respectively. For Fv/Fm, the Soil model ranked first, although the Base model was competitive (ΔAICc = 0.75) and both had negative LOO R^2^ values. Accordingly, the current data support moderate out-of-sample prediction only for plant height, not for crown or physiological traits.

## 4. Discussion

### 4.1. Edaphic Differentiation Was Stronger than Field Trait Differentiation

The principal finding was a pronounced asymmetry between environmental and functional differentiation. Within Tarbagatai, species identity explained 48.6% of variation in the four-variable soil matrix but only 9.2% of variation in the plant-trait matrix. This contrast persisted after aggregation to species–site units and was supported by omnibus differences in soil pH, EC, TN, and TC. Dunn’s tests showed that the clearest conservative pairwise contrasts involved pH and EC, together with the TN–TC separation of *R. acicularis* and *R. alberti*. By comparison, height, crown width, maximum PSII efficiency, CFR, and estimated chlorophyll overlapped substantially among species.

These results indicate that ecological differentiation among the studied wild roses was expressed more clearly in the edaphic environments they occupied than in their realised morphology or photosynthetic state at sampling. The pattern is consistent with environmental filtering, whereby distributions are constrained by soil chemistry, moisture regime, nutrient availability, microbial activity, and associated habitat characteristics [60,61,62]. Environmental filtering need not produce complete divergence in readily measured above-ground traits; related taxa may occupy different environmental niches while maintaining overlapping functional states through acclimation, compensatory trait combinations, or selection of favourable microsites [8,9,63].

The soil PERMDISP result indicates that the PERMANOVA signal reflected both separation of species centroids and differences in the breadth of the soil conditions occupied by each species. This distinction is ecologically informative: *R. alberti* was associated with a relatively restricted set of low-EC, low-TN, and low-TC sites; *R. spinosissima* occupied a broader range; and *R. acicularis* was concentrated in more acidic, carbon- and nitrogen-enriched soils. The species may therefore differ in both edaphic niche position and niche breadth.

The direction of separation was internally consistent. Soil pH influences nutrient solubility, cation exchange, microbial composition, decomposition, and root nutrient acquisition [29,64]. Higher TN and TC near *R. acicularis* may reflect greater organic-matter accumulation, litter input, or moisture retention. In contrast, lower TN, TC, and EC near *R. alberti* may reflect substrates that are more open, weakly developed, erosion-prone, or rapidly drained. These mechanisms remain hypotheses because texture, carbonates, depth, moisture, litter mass, slope position, and canopy cover were not measured.

The correlation structure also shows why TN, TC, and EC should not be interpreted as independent causal drivers. TN and TC were almost perfectly correlated, and both increased with EC. Soil PC1, therefore, provides a more defensible summary of the measured edaphic structure than separate causal interpretations. The pattern may also partly reflect plant-mediated rhizosphere modification through litter inputs, root exudation, nutrient uptake, and interactions with soil biota [60,61,62]. Distinguishing habitat filtering from plant-driven soil modification will require paired sampling beneath and outside shrub canopies and reciprocal soil-inoculation experiments.

### 4.2. Similar Field Physiology Does Not Imply Ecological Equivalence

The absence of pronounced interspecific differences in chlorophyll fluorescence and chlorophyll-related variables does not imply that the three species share identical physiological strategies. It shows only that their realised photosynthetic states overlapped during the limited period and environmental conditions represented by the field measurements.

Mean Fv/Fm values were similar among species. Although Fv/Fm is widely used to evaluate maximum PSII photochemical efficiency, it is influenced by dark-adaptation duration, previous irradiance, leaf age, temperature, water status, phenology, nutrient status, and time of day [14,15,16]. A single field campaign, therefore, provides a snapshot of current leaf performance rather than a comprehensive measure of drought tolerance, nutrient-use efficiency, or long-term stress resistance.

Plants in contrasting habitats can converge in instantaneous photosynthetic performance despite differences in rooting depth, stomatal regulation, osmotic adjustment, leaf turnover, allocation, or phenology. Phenotypic plasticity may enable established shrubs to maintain similar function after strong environmental filtering [7,8,9,63]. Field surveys also sample surviving adults rather than seedlings that failed to recruit; differentiation may act most strongly during germination, establishment, winter survival, or extreme drought.

The strong CFR–chlorophyll association was expected because both values originate from the same CCM-300 signal. It confirms internal consistency but does not provide two independent biological responses. This redundancy was therefore removed from the PCA. A fuller physiological assessment should combine fluorescence with gas exchange, water potential, stomatal conductance, tissue nutrient levels, stable isotopes, osmotic adjustment, and oxidative stress markers.

The absence of FDR-supported plant–soil correlations and the non-significant partial redundancy analysis indicate that simple one-to-one relationships with the four soil variables did not govern the measured plant responses. This does not show that soils were biologically unimportant; rather, the available soil panel was insufficient to predict the selected traits linearly at the sampled spatial and temporal scale.

### 4.3. Regional Responses Were Species Specific

Regional differentiation was species-specific. *R. spinosissima* showed the clearest above-ground pattern, with taller individuals in the Altai than in Tarbagatai at the observation level. The effect was large, although it became non-significant after aggregation and multiplicity correction, indicating that its direction was consistent but its statistical support depended partly on repeated observations.

Greater height in the Altai could reflect higher moisture availability, deeper soils, a more favourable effective growing season, reduced exposure, stronger competition for light, lower grazing pressure, disturbance history, or population age structure. It could also reflect genetic differentiation. Field observations cannot separate plasticity from inherited reaction norms; common-garden and reciprocal-transplant experiments are required to test persistence under uniform conditions and local adaptation through relative fitness [65,66,67].

For *R. acicularis*, regional differentiation was stronger in the soil matrix than in the plant-trait matrix. This decoupling may reflect functional buffering, plastic adjustment in unmeasured traits, or the occupation of comparable microsites within contrasting landscapes. Still, it may also arise from limited power or seasonal convergence. The contrasting responses of *R. acicularis* and *R. spinosissima* show that mountain-system identity should not be treated as a universal predictor across the genus.

### 4.4. Geographic Structure and Limits of Prediction

Model comparison supported a conservative interpretation. Species identity and mountain system provided a moderate, partly cross-validated signal for plant height, whereas adding soil PC1 and local coordinates did not improve prediction. Cross-validated performance for crown width, Fv/Fm, CFR, and chlorophyll was weak or negative.

Ecologically, the weak predictive performance indicates that the measured traits cannot be assigned reliable values based on species, region, available soil gradient, or local coordinate position alone. Short-term physiology and crown dimensions were likely shaped by unmeasured factors, including microclimate, soil moisture, phenology, ontogeny, competition, disturbance, and local history. The models should therefore not be used to generate management thresholds or deterministic trait maps. Their value is instead diagnostic: they show that the current predictors captured a repeatable component of stature but little transferable information about instantaneous physiological state.

Local northing and easting are broad proxies rather than mechanistic variables. Small coordinate shifts may correspond to large differences in elevation, aspect, cold-air drainage, snow persistence, soil depth, canopy cover, or distance from water. At the same time, distant sites may contain similar microsites. Future spatial analyses should use verified predictors of elevation, terrain, moisture, climate, vegetation, and disturbance, and should employ grouped or spatially blocked validation by independent locality [54,55].

The results also illustrate the distinction between explanatory fit and predictive performance. Additional predictors may improve in-sample fit yet reduce generalisability in small ecological datasets. The near-zero or negative LOO R^2^ values provide direct evidence that most apparent effects were not stable enough for broader prediction.

### 4.5. Conservation Implications and Future Research

The conservation relevance of these findings lies in the environmental heterogeneity represented by wild *Rosa* occurrences. Geographic records alone do not capture the full edaphic breadth of natural populations, and collections concentrated in accessible or environmentally similar sites may underrepresent plastic or adaptive variation.

Conservation sampling should therefore be stratified by species, mountain system, and soil context rather than based solely on geographic distance or administrative boundaries. *R. acicularis* collections should include acidic, carbon- and nitrogen-rich sites and less enriched soils; the preliminary *R. alberti* pattern highlights low-EC, nutrient-poor sites; and the broad edaphic envelope of *R. spinosissima* supports sampling across multiple contrasting environments. In situ and ex situ passport data should include soil chemistry, elevation, slope, aspect, vegetation, moisture, population size, associated species, and disturbance [68].

The present results should not be used to classify populations as stress-tolerant or locally adapted. A rigorous next step is a factorial common-garden experiment using populations spanning contrasting soil PC1 values and controlled pH, nutrient, salinity or conductivity, and water treatments. Reciprocal experiments with sterilised and non-sterilised home and foreign soils could separate abiotic soil effects from microbial feedbacks. Coupling these designs with genomic or reduced-representation sequencing would permit tests of population structure and genotype–environment association.

### 4.6. Study Limitations

Several limitations define the scope of inference. The design was unbalanced: *R. alberti* was represented by only 4 records from one mountain system, and sampling covered only one field season. Some records shared coordinates, while archived metadata were incomplete for shrub identity, biological replication, soil compositing and depth, leaf position, and dark-adaptation duration. Aggregation reduced but could not eliminate the consequences of clustered sampling.

The edaphic panel was restricted to pH, EC, TN, and TC and omitted soil moisture, texture, bulk density, available nutrients, carbonates, cation-exchange capacity, rooting depth, microbial composition, and mycorrhizal colonisation. The significant soil PERMDISP result also indicates that species separation included differences in environmental variance and centroid position.

Finally, the observational design precludes causal and adaptive inference. Species may respond to soil conditions, modify them, or covary with unmeasured environmental factors. Nevertheless, the convergence of univariate, species–site, multivariate, correlation, and model-validation results supports the central field pattern: environmental segregation was stronger and more reproducible than divergence in the measured realised traits.

## 5. Conclusions

Wild populations of *R. acicularis*, *R. alberti*, and *R. spinosissima* in eastern Kazakhstan were differentiated more strongly by the edaphic conditions of their habitats than by morphological and ecophysiological traits measured during a single field campaign. Within Tarbagatai, species identity explained substantial multivariate soil variation, whereas plant-trait separation was weak and conditional plant–soil covariation was non-significant. A regional–taxonomic signal remained for plant height, particularly in *R. spinosissima*, but models augmented with soil and coordinate predictors did not improve out-of-sample prediction for most traits.

The results support edaphic niche differentiation, accompanied by limited divergence in realised field trait states, but do not demonstrate physiological or local adaptation. They provide a basis for environmentally stratified conservation sampling and for common-garden, reciprocal-transplant, soil-manipulation, and repeated-monitoring experiments designed to test plasticity, tolerance, plant–soil feedbacks, and heritable adaptation.

## Figures and Tables

**Table 1:** Sampling distribution by species and mountain system.

Species	Mountain System	Observations (*n*)	Unique Coordinates (*n*)	Species–Site Units (*n*)
*Rosa acicularis*	Altai	4	4	4
*Rosa acicularis*	Tarbagatai	11	8	8
*Rosa alberti*	Altai	0	0	0
*Rosa alberti*	Tarbagatai	4	4	4
*Rosa spinosissima*	Altai	7	7	7
*Rosa spinosissima*	Tarbagatai	14	7	7
Total	—	40	25	30

Note: Species–site units are unique combinations of species and geographic coordinates used in aggregated analyses.

**Table 2:** Descriptive statistics by species (observation-level means ± standard deviations).

Variable	*R. acicularis* (*n* = 15)	*R. alberti* (*n* = 4)	*R. spinosissima* (*n* = 21)
Height (cm)	73.6 ± 31.6	59.7 ± 6.9	91.6 ± 31.7
Crown width (cm)	69.8 ± 37.9	100.3 ± 26.7	65.9 ± 44.0
Fv/Fm	0.761 ± 0.017	0.739 ± 0.068	0.747 ± 0.029
Fv/Fo	3.206 ± 0.277	2.996 ± 0.887	3.011 ± 0.570
CFR (F735/F700)	1.191 ± 0.085	1.159 ± 0.053	1.203 ± 0.096
Chlorophyll (mg m^−2^)	368.3 ± 45.0	349.0 ± 38.1	366.2 ± 60.7
Soil pH	5.08 ± 0.36	5.55 ± 0.14	5.41 ± 0.40
EC (µS cm^−1^)	250.4 ± 90.3	123.3 ± 29.0	270.3 ± 115.9
Total nitrogen (%)	0.78 ± 0.32	0.31 ± 0.06	0.46 ± 0.15
Total carbon (%)	9.54 ± 4.65	3.33 ± 0.92	5.19 ± 1.67

**Table 3:** Kruskal–Wallis tests among species within Tarbagatai at the observation level and after aggregation to species–site means.

Variable	*H* (Obs.)	*q* (Obs.)	ε^2^ (Obs.)	*H* (Site)	*q* (Site)	ε^2^ (Site)
Height (cm)	3.69	0.192	0.065	3.84	0.294	0.115
Crown width (cm)	4.34	0.179	0.090	0.98	0.611	0.000
Fv/Fm	4.16	0.179	0.083	2.15	0.413	0.009
Fv/Fo	4.16	0.179	0.083	2.15	0.413	0.009
CFR (F735/F700)	3.30	0.192	0.050	2.43	0.413	0.027
Chlorophyll (mg m^−2^)	3.47	0.192	0.056	1.98	0.413	0.000
Soil pH	9.99	0.023	0.307	8.49	0.037	0.406
EC (µS cm^−1^)	7.62	0.055	0.216	8.43	0.037	0.402
Total nitrogen (%)	12.11	0.012	0.389	11.42	0.021	0.589
Total carbon (%)	12.09	0.012	0.388	10.90	0.021	0.556

Notes: Obs., 40 observation records; Site, 30 species–site means. *q* values were adjusted across the ten omnibus tests at each analytical resolution.

**Table 4:** Dunn post hoc comparisons of soil variables among species within Tarbagatai using species–site means.

Variable	Comparison	Median 1	Median 2	Dunn *z*	Adjusted *q*
Soil pH	*acicularis* vs. *alberti*	5.26	5.56	−2.43	0.023
Soil pH	*acicularis* vs. *spinosissima*	5.26	5.53	−2.44	0.023
Soil pH	*alberti* vs. *spinosissima*	5.56	5.53	0.36	0.715
EC (µS cm^−1^)	*acicularis* vs. *alberti*	248.4	115.0	2.72	0.017
EC (µS cm^−1^)	*acicularis* vs. *spinosissima*	248.4	232.5	0.14	0.888
EC (µS cm^−1^)	*alberti* vs. *spinosissima*	115.0	232.5	−2.54	0.017
Total nitrogen (%)	*acicularis* vs. *alberti*	0.65	0.31	3.30	0.003
Total nitrogen (%)	*acicularis* vs. *spinosissima*	0.65	0.39	1.96	0.074
Total nitrogen (%)	*alberti* vs. *spinosissima*	0.31	0.39	−1.61	0.108
Total carbon (%)	*acicularis* vs. *alberti*	7.69	3.13	3.19	0.004
Total carbon (%)	*acicularis* vs. *spinosissima*	7.69	4.66	2.04	0.063
Total carbon (%)	*alberti* vs. *spinosissima*	3.13	4.66	−1.44	0.150

Notes: Adjusted *q* values are Benjamini–Hochberg corrections within each variable. The group order determines the sign of *z*.

**Table 5:** Species–site PERMANOVA and multivariate-dispersion results.

Comparison	Response Matrix	df	*F*	*p*	R^2^	PERMDISP *p*
Species: *Tarbagatai*	plant	2, 16	0.81	0.5761	0.092	0.6569
Species: *Tarbagatai*	soil	2, 16	7.57	0.0005	0.486	0.0443
Species: *Tarbagatai*	combined	2, 16	2.91	0.0015	0.267	0.7283
Region: *R. acicularis*	plant	1, 10	1.58	0.1919	0.137	0.7749
Region: *R. acicularis*	soil	1, 10	3.57	0.0330	0.263	0.6447
Region: *R. acicularis*	combined	1, 10	2.39	0.0299	0.193	0.8264
Region: *R. spinosissima*	plant	1, 12	3.06	0.0272	0.203	0.3772
Region: *R. spinosissima*	soil	1, 12	2.04	0.1297	0.145	0.0034
Region: *R. spinosissima*	combined	1, 12	2.59	0.0180	0.177	0.5434

**Table 6:** Selected pairwise correlations at the observation and species–site levels.

Factor Pair	ρ (Obs.)	*q* (Obs.)	ρ (Site)	*q* (Site)	Linear R^2^ (Site)
TN–TC	0.986	<0.001	0.989	<0.001	0.965
EC–TN	0.485	0.011	0.611	0.003	0.174
EC–TC	0.498	0.011	0.613	0.003	0.145
pH–TN	−0.491	0.011	−0.447	0.096	0.141
pH–TC	−0.473	0.012	−0.432	0.103	0.101
Height–EC	0.419	0.035	0.416	0.114	0.268
Height–width	0.415	0.035	0.397	0.135	0.170
CFR–chlorophyll	0.935	<0.001	0.892	<0.001	0.804

Notes: *q* values were adjusted across all 45 pairwise tests. CFR and estimated chlorophyll are not independent because the chlorophyll estimate is instrument-derived from CFR.

**Table 7:** Complete candidate-model comparison for five standardised plant responses.

Response	Model	AICc	ΔAICc	Akaike Weight	Adjusted R^2^	LOO R^2^
Plant height	Base	−7.81	0.00	0.748	0.376	0.294
Plant height	Soil	−5.18	2.64	0.200	0.356	0.211
Plant height	Spatial	−2.09	5.73	0.043	0.331	0.260
Plant height	Full	1.09	8.90	0.009	0.308	0.171
Crown width	Base	4.66	0.00	0.766	0.054	−0.090
Crown width	Soil	7.49	2.84	0.186	0.018	−0.170
Crown width	Spatial	10.52	5.86	0.041	−0.018	−0.168
Crown width	Full	13.89	9.23	0.008	−0.060	−0.258
Fv/Fm	Base	7.77	0.75	0.388	−0.049	−0.400
Fv/Fm	Soil	7.02	0.00	0.564	0.034	−0.373
Fv/Fm	Spatial	13.46	6.44	0.023	−0.123	−0.999
Fv/Fm	Full	13.24	6.22	0.025	−0.037	−0.917
CFR	Base	2.53	0.00	0.725	0.119	−0.039
CFR	Soil	5.04	2.50	0.207	0.095	−0.110
CFR	Spatial	7.68	5.14	0.055	0.074	−0.245
CFR	Full	10.77	8.24	0.012	0.044	−0.337
Chlorophyll	Base	0.35	0.00	0.659	0.180	0.023
Chlorophyll	Soil	2.00	1.64	0.290	0.183	−0.008
Chlorophyll	Spatial	6.06	5.71	0.038	0.122	−0.319
Chlorophyll	Full	8.28	7.92	0.013	0.121	−0.400

Notes: Base = species + mountain system; Soil = Base + soil PC1; Spatial = Base + local northing + local easting; Full = Base + soil PC1 + local coordinates. Negative LOO R^2^ indicates performance worse than prediction by the held-out mean.

## Data Availability

The original contributions presented in this study are included in the article. Further inquiries can be directed to the corresponding authors.
